# Pest mites and their interaction with Phytoseiidae and other arthropod predators in an almond orchard in South-West Spain

**DOI:** 10.1007/s10493-022-00746-3

**Published:** 2022-11-03

**Authors:** José E. González-Zamora

**Affiliations:** Department of Agronomía, Universidad de Sevilla - Carretera de Utrera, km 1, 41013 Sevilla, Spain

**Keywords:** Spider mites, Almond, Predators, *Euseius stipulatus*, *Typhlodromus* (*Anthoseius*) *athenas*

## Abstract

The almond crop in Spain has increased in importance in recent years and consequently there is a need to improve knowledge about pests, diseases, and weeds. The present study was conducted from 2017 to 2020, with the objective of determining the fauna of spider mites and their natural enemies, with a special emphasis on phytoseiids. The main spider mite species was *Tetranychus urticae* Koch, and secondary species were *Bryobia rubrioculus* (Scheuten) and *Eutetranychus banksii* (McGregor). Phytoseiidae were the most abundant group of natural enemies, with 59% of the individuals observed; *Euseius stipulatus* (Athias-Henriot) was the predominant species, accounting for 96% of adult females identified, *Typhlodromus* (*Anthoseius*) *athenas* Swirski & Ragusa accounted for the remaining 4%. Other (potential) natural enemies were Chrysopidae, *Scolothrips longicornis* Priesner, and *Stethorus* sp. with 36, 2, and 3%, respectively, of the natural enemy individuals. The seasonal pattern of *T. urticae* indicated population peaks from July to September, and its control was based on miticides in most seasons. *Euseius stipulatus* and *T. athenas* appeared mainly in May–June and did not show interaction with the spider mite population. Chrysopidae were present throughout the season, from May to October in the 4 years, but no direct relationship with the spider mite population was observed. In contrast, the seasonal pattern of both *S. longicornis* and *Stethorus* sp. coincided with the most important peaks of spider mites and these predators were seen in the spider mite colonies, although in very low numbers. The importance of these latter specialized spider mite predators and ways to strengthen them are discussed.

## Introduction

The almond crop acreage in Spain has increased in recent years, by about 36.3% between 2014 and 2020, reaching 718,540 ha in the last available statistics (MAPA 2015, 2020), stimulated by the good prices for fruit and future prospects (MAPA 2019). This increase occurred mainly in new plantations that replaced other less profitable crops in areas with irrigation rights (i.e., relatively dry areas that were given the right to irrigate crops; Junta de Andalucia 2016), which means an increase in irrigated almond acreage of 153% (118,202 ha in 2020, around 25% in Andalucia; MAPA 2015, 2020).

Almond pests (and diseases) were little studied in Spain until these recent changes, but new interest has led to studies on how they affect the crop in this new situation (Sánchez-Ramos et al. 2015; Ollero-Lara et al. 2016, 2019; Torguet Pomar et al. 2016; Durán et al. 2017b). The main pests of almond orchards in Spain are aphids [mainly *Hyalopterus amygdali* (Blanchard)] and mites [especially *Tetranychus urticae* Koch (Trombidiformes: Tetranychidae), but secondary species are *Bryobia rubrioculus* (Scheuten) and *Eutetranychus* spp.]; other groups may be relevant in particular locations and moments, such as certain Hemiptera [*Asymmetrasca decedens* (Paoli), *Monosteira unicostata* (Mulsant and Rey), *Parlatoria oleae* (Colvée)], and sometimes Coleoptera [*Capnodis tenebrionis* (L.)] and Lepidoptera (*Anarsia lineatella* Zeller). Various pathogens affect almond crops, although the crop variety and management strategy influence damage severity (Ollero-Lara et al. 2019). In other areas where almonds are of particular importance, such as California (USA), studies on the main pests and diseases of the crop have been carried out for a long time (Haviland et al. 2022). Briefly, the most important pest species in California almonds are *Amyelois transitella* (Walker) (Lepidoptera: Pyralidae) (Shorey and Gerber 1996; Wilson et al. 2020; Haviland et al. 2021a) and spider mites, mainly the species *Tetranychus pacificus* McGregor and *T. urticae* (Welter et al. 1984; Wilson et al. 1984; Haviland et al. 2022). Regarding tetranychids, there is much interest in the presence and effect of natural enemies of spider mites, focused initially on phytoseiids (Mesostigmata: Phytoseiidae) although recently also other groups have attracted interest (Hoy et al. 1979; Grafton-Cardwell et al. 2020; Haviland et al. 2021b).

This detailed knowledge of mite species and their predators in Californian almonds contrasts with the Spanish situation – no specific study on the presence of the main predators of spider mites on almond has been carried out, and no mention was made of any survey of Phytoseiidae mites on almond in specialized literature (Ferragut et al. 2010). On the other hand, almond is considered a drought-tolerant species and its response to water scarcity has been defined in many studies under deficit irrigation (Gutiérrez-Gordillo et al. 2019; Martín-Palomo et al. 2019; García-Tejero et al. 2020), which can be considered a necessity in the near future in more arid scenarios, as happens in the Mediterranean basin (EEA 2017). Following the new tendencies in water management, a recent study (González-Zamora et al. 2021) indicated that a deficit irrigation regime does not affect the abundance and seasonal pattern of phytoseiids and spider mites, but the damage inflicted by the latter on leaves is less severe in a deficit irrigation system compared to a more irrigated treatment.

The present study aims at characterizing the presence of spider mite pests in an almond orchard during a study that lasted from 2017 to 2020, and the predator fauna associated, with special emphasis on phytoseiid species, seasonal patterns, and relationship with the pests, but also including any other (potential) natural enemy that was found.

## Materials and methods

### Experimental design

The surveys were carried out in an orchard in Dos Hermanas (province of Sevilla, Spain; 37°13.805´N, 5°54.823’W). It has an area of 29,423 m^2^, and the survey was carried out on 7,968 m^2^. The orchard had two cultivated almond [*Prunus dulcis* (Mill) DA Webb, Rosaceae] varieties, ‘Vairo’ and ‘Guara’, planted in paired lines, with a tree spacing of 6 × 8 m; the surveys were carried out only on the cultivar ‘Vairo’. The trees were 7 years old at the beginning of the experiment in 2017, which lasted until 2020. The orchard was fertilized and controlled for pests, diseases, and weeds using the criteria of the owner and advisor technicians. The timing and products applied in the 4 years are listed in Appendix 1. Sampling was in principle biweekly, but actual samplings were performed several days before or after the application schedule, to limit contact with residues.

The experiment had a complete randomized block design, with four blocks and two irrigation treatments. Each experimental plot had 12 trees (four rows with three trees each), with the two central trees (cv. Vairo) in each plot used for sampling purposes. A repetition of each irrigation treatment was randomly assigned within each block, making four repetitions of each irrigation treatment and eight plots for the entire experiment. The present study analyzes the presence of mite pests, phtytoseiidae mites and other natural enemies using the eight replicates.

### Sampling procedure

Samplings were carried out from March to September/October in each year of the study, except in 2020, when sampling started in mid-May when COVID-19 pandemic lockdown restrictions were relaxed. Samplings were (in principle) performed biweekly, with 18 dates in 2017, 18 in 2018, 13 in 2019, and 14 in 2020.

The two central trees of each plot or repetition were scouted and two shoots (each around 6 cm, with three–four leaves) were randomly selected in each cardinal direction per tree (16 branches per plot) and visually observed for mites and other arthropods, with a total of 128 branches on each sampling date. Leaf area damage produced by spider mites was estimated using an ordinal scale: 0 (no damage); 1 (1–20% of surface damaged); 2 (21–50% of surface damaged); 3 (> 50% of surface damaged). Means per plot were calculated, and average values and standard errors were calculated based on the eight plots per sampling date.

Visual sampling was carried out mainly with direct counts of mites and insect individuals on the shoots, except for spider mites in 2017 – the first year of the survey – in which presence/absence was used.

Shoots with mite pests and arthropods were taken to the laboratory to identify the species, and specifically with phytoseiids, they were directly collected in the field with a brush and introduced in vials with 70% ethanol. Mite specimens were cleared in lactic acid at 45–50 ºC for 24–48 h and mounted in Hoyer’s medium until their identification with a Nikon Labophot-2 microscope at 400× magnification. The specimens were separated following various generic taxonomic guides (Chinery 1997; Barrientos 2004) and specific keys for thrips and mites were used (Jeppson et al. 1975; Mound et al. 1976; Ferragut and Santonja 1989; Ferragut et al. 2010; Vacante 2016). Slides of the most relevant specimens are kept at the laboratory collection.

## Results

**Fig. 1:**
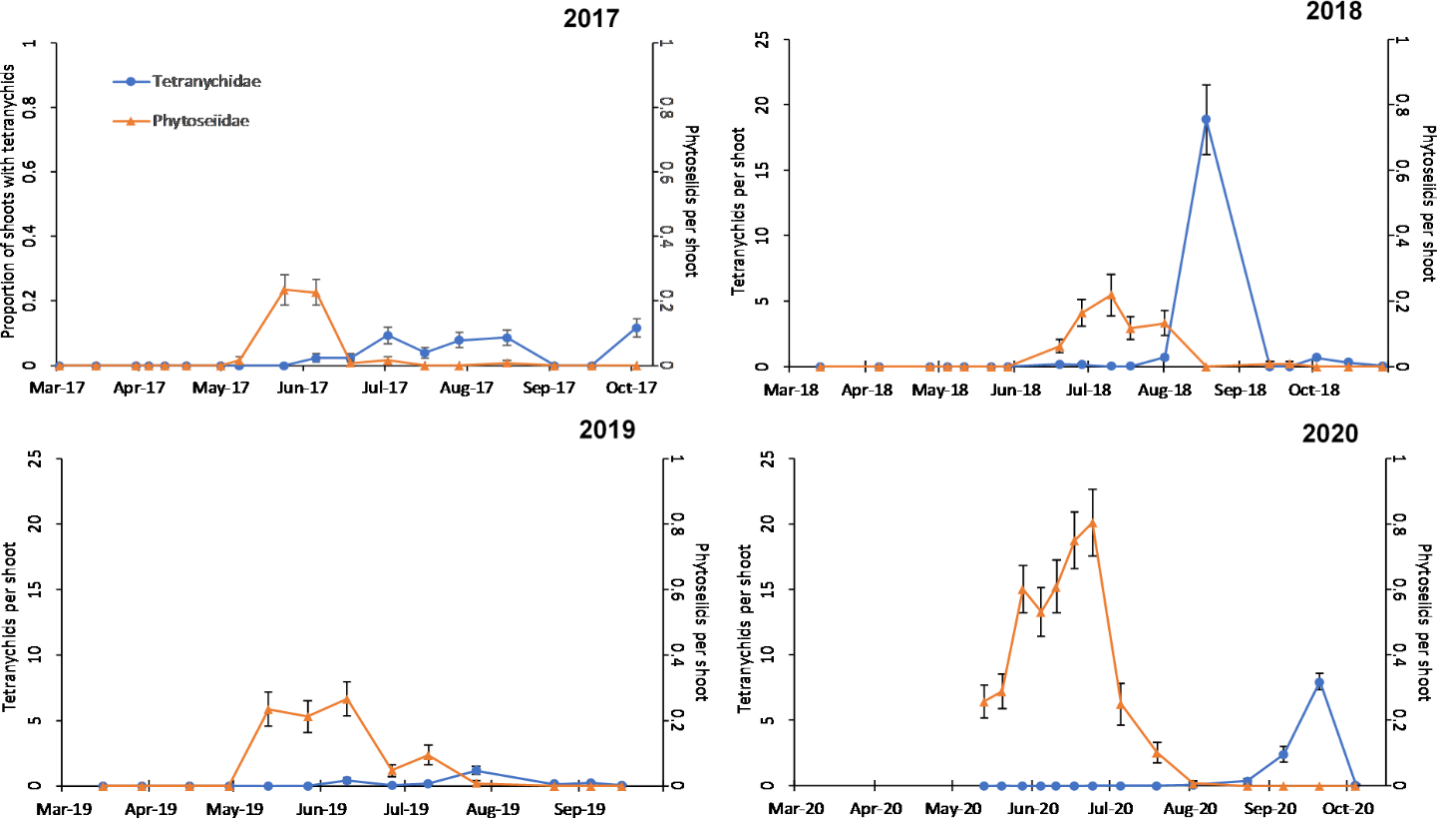
Seasonal pattern of Tetranychidae (*Tetranychus* spp., with the presence of individuals of *Eutetranychus banksii* at the end of the season in 2019 and 2020) in proportion of almond shoots occupied (2017) and in population density in shoots (2018–2020) together with population density of Phytoseiidae on shoots in the 4 years of study. The error bars indicate the exact 95% confidence intervals of the proportion of Tetranychidae in 2017, and the standard errors around the mean values for the remainder

Tetranychid mites were regularly present in the almond orchard during the 4 years of the study (Table 1), their densities varying throughout the study: their presence was low in 2017 (10% of shoots occupied) and 2019 (in total 359 individuals sampled) (Fig. 1; Table 1), with moderate leaf damage (from 5 to near 20% of leaf area; see González-Zamora et al. 2021), whereas in 2018 and 2020 the numbers were much higher (2,694 and 1,455 individuals, respectively; Table 1) and caused consideraly leaf damage (from 20 to 60% of leaf area; see González-Zamora et al. 2021).

**Table 1:** Total numbers of specimens of the most abundant phytophagous and predaceous Acari and the principal natural enemies of mites observed in the 4 years of the study in an almond orchard. Total number of shoots observed: 2,304 (2017), 2,304 (2018), 1,664 (2019), 1,792 (2020)

	2017		2018		2019		2020		Total
Tetranychidae	—		2,694		359		1,455		4,508
*Tetranychus* spp.	—^1^		2,687		285		1,388		4,360
*Eutetranychus banksii*	—^2^		—^2^		64		57		121
*Bryobia rubrioculus*	—^2^		7		10		10		27
Phytoseiidae	65		91		110		538		804
Chrysopidae^3^	186		136		70		98		490
*Scolothrips longicornis*	5		7		0		16		28
*Stethorus* sp.	2		0		7		32		41

^1^ Population was determined as presence/absence

^2^ Population was not determined due its low numbers

^3^ Counts were mainly of eggs

The seasonal pattern of tetranychids shows that their densities are higher in the middle and end of the summer, with peaks in August 2018, July 2019, and September 2020, reaching near 19, one, and eight individuals per shoot, respectively; Fig. 1). Also in 2017 tetranychid numbers were higher in the second half of summer, with no clear peak in density (Fig. 1).

The most abundant tetranychid species identified was *T. urticae*, although a rigorous species determination was made only in 2020 (Table 2). Other species were also observed in association, such as *Tetranychus turkestani* Ugarov & Nikolskii (Table 2, but only one male identified in 2020), *Eutetranychus banksii* (McGregor), present at the end of the season, and *B. rubrioculus*, which appeared in April–May, but in low numbers (Tables 1 and 2).

**Table 2:** Total number of mite specimens mounted on slides for identification during the 4 years of the study in the almond orchard

		2017		2018		2019		2020		Total
Tetranychidae										59
*Tetranychus urticae*	Males	1		‒		‒		11		12
*Tetranychus turkestani*	Males	‒		‒		‒		1		1
*Tetranychus* sp.	Males	‒		‒		‒		18		18
	Females	2		3		4		4		13
	Juveniles	‒		1		‒		‒		1
*Eutetranychus banksii*	Females	‒		‒		6		‒		6
*Bryobia rubrioculus*	Females	‒		6		2		‒		8
Phytoseiidae										167
*Euseius stipulatus*	Females	10		17		31		87		145
*Typhlodromus athenas*	Females	1		4		1		‒		6
Other specimens	Males	‒		2		3		7		12
	Juveniles	1		‒		3		‒		4

Phytoseiids were observed during the 4 years of the study (Table 1), with a varying abundance among years (highest total number was 538 individuals in 2020). They were observed in higher quantities at the end of spring and the beginning of summer in the 4 years (May to June-July, Fig. 1), with peaks of ca. 0.2 individuals per shoot (0.8 in 2020); they were almost absent during the rest of the season, showing no relation at all with the tetranychids seasonal pattern (Fig. 1). *Euseius stipulatus* (Athias-Henriot) was the most abundant species identified (Table 2), with 96% of adult females belonging to this species. *Typhlodromus* (*Anthoseius*) *athenas* Swirski & Ragusa accounted for 4% of the recovered females (Table 2).

**Fig. 2:**
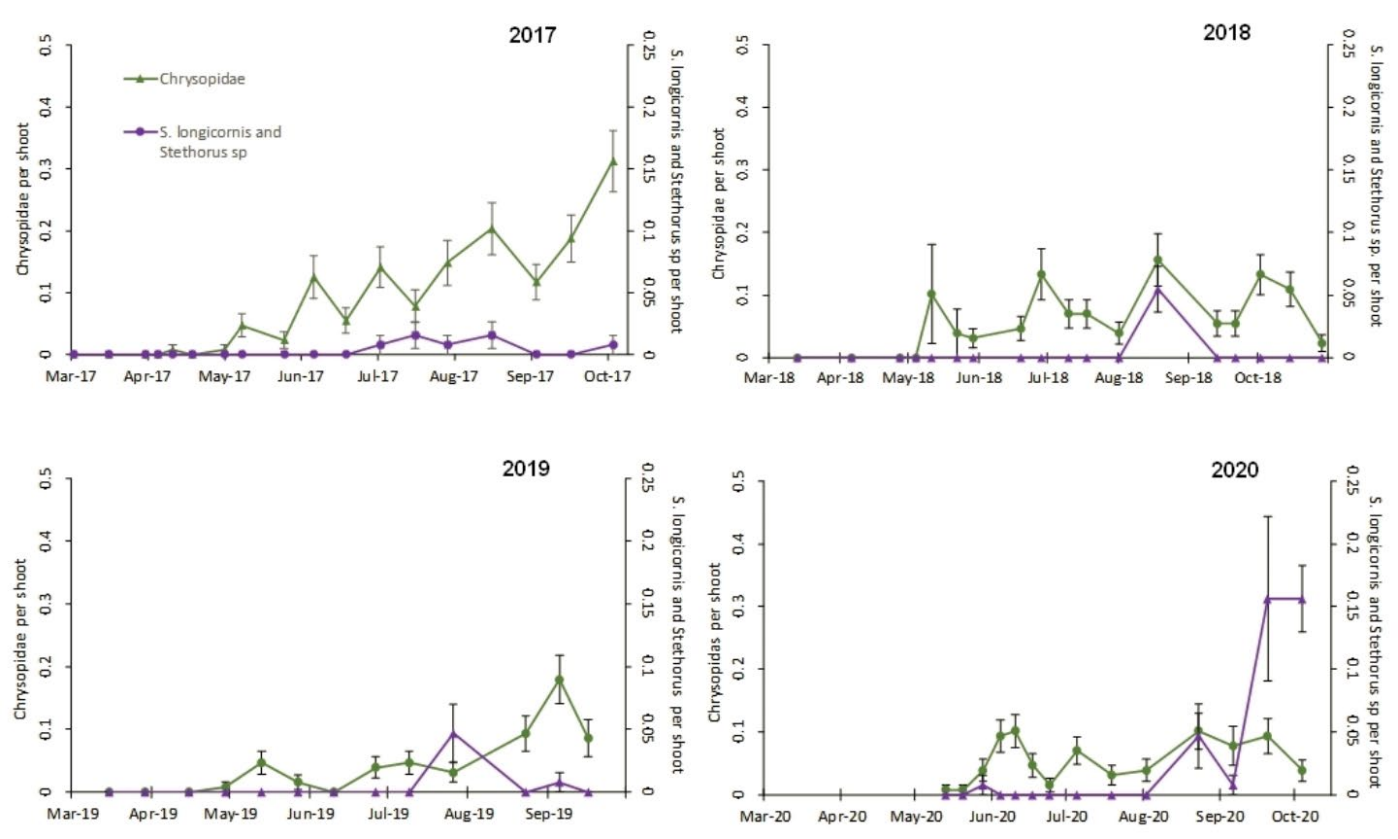
Seasonal pattern of densities (mean ± SE number per shoot) of Chrysopidae and *Scolothrips longicornis* plus *Stethorus* sp. in an almond orchard in 2017–2020

Insect predators were also observed during the sampling period. Chrysopidae (Neuroptera; no species were identified) were the most abundant during the study, varying between 70 and 186 individuals per year in total (mainly eggs, Table 1), although their population trends were quite independent of the presence of tetranychids, with mean numbers around 0.1–0.2 individuals per shoot (Fig. 2). Specific predators of tetranychids were observed in small quantities (Table 1): 28 individuals of *Scolothrips longicornis* Priesner (Thysanoptera: Thripidae) and 41 of *Stethorus* sp. (Coleoptera: Coccinellidae), both of which appeared during the peaks of the tetranychids population during the 4 years of the study (Fig. 2).

## Discussion

The Tetranychidae population showed different seasonal patterns between the years of study. They did not cause any particular problem in 2017 and 2019. The seasonal pattern showed a clear population peak in August–September in 2018 and 2020, which is usually observed in Andalucía (Durán et al. 2017a) as well as in California, USA (Tollerup and Higbee 2020; Haviland et al. 2022). After the peak, a quick reduction is observed, which may be due to the presence of predators, miticide application, abiotic conditions, or any combination of these factors. Such population peaks provoked evident foliar damage. The owner tried to regulate the mite population by applying miticides during the four seasons (see Appendix 1), with, for example, only one miticide application in 2020. *Tetranychus urticae* is probably the predominant spider mite in the orchard along the study, although a rigorous species determination was made only in 2020. Related species, such as *T. turkestani*, was also detected. *Tetranychus urticae* is the most important tetranychid species reported in Spanish almond crops, although other mites present are *B. rubrioculus*, *Eutetranychus orientalis* (Klein), *E. banksii*, *Eotetranychus carpini* Oudemans, and *Panonychus ulmi* (Koch) (Martín Gil et al. 2015; Durán et al. 2017a). In California almonds, *Tetranychus pacificus* McGregor and *T. urticae* are the most abundant spider mite pests (Haviland et al. 2021d*urticae* and *Schizotetranychus smirnovi* Wainstein are the most abundant spider mite in almond in Iran (Saeidi and Nemati 2020). In all cases the seasonal pattern is very similar, with peak populations in summer and subsequent leaf damage, which, if severe, causes defoliation that leads to reductions in tree development and yield the following year (Welter et al. 1984).

*Euseius stipulatus* was the most abundant phytoseiid in the orchard during the 4 years of this study. It is also one of the most frequent and abundant phytoseiid species in Spain, very common in citrus (Ferragut and Escudero 1997; Abad-Moyano et al. 2009) and other crops (Ferragut and Escudero 1997; Ferragut et al. 2010). No previous study on – or reference to – its presence in almonds in Spain has been made.

Phytoseiid species were reported in almonds many years ago in California (Rice and Jones 1978; Hoy et al. 1979), highlighting the importance of *Galendromus occidentalis* (Nesbitt) in regulating *Tetranychus* species. However, recent studies reflect the diversity of species and the change in composition of the phytoseiid fauna that can be found in different California crops: six phytoseiid species were recorded on almond, with 16% of the individuals belonging to *E. stipulatus* (and 30% to *Euseius* spp.) (Grafton-Cardwell et al. 2020). These authors also suggested that the previously most important spider mite predator in almond has shifted to other species of arthropods, as several studies have indicated the thrips *Scolothrips sexmaculatus* Pergande as currently the most relevant predator of spider mites, together with *Stethorus punctum* LeConte (Tollerup and Higbee 2020; Haviland et al. 2021b, c). This change in the fauna of spider mite predators is considered a consequence of recent changes in the California almond industry, particularly the elimination of dormant and in-season organophosphate insecticide use (Haviland et al. 2021b), which allows the resurgence and predominance of better-adapted insect predators of spider mites (and also of other mites as phytoseiids are), but that are more susceptible to such pesticides.

*Euseius stipulatus* is regarded as a type IV species (McMurtry and Croft 1997; McMurtry et al. 2013), that feeds on pollen and several arthropods, some of them pests of interest such as the non-web-producing mite *Panonychus citri* (McGregor) in citrus crop (García-Marí 2012), but of lesser interest for the control of other agricultural pests, especially tetranychids that produce a dense web, such as *T. urticae* (Ferragut et al. 1987). The *E. stipulatus* population in the orchard increases at the end of May, but the almond bloom finishes at the end of March, so this increment cannot rely solely on the mites feeding on pollen that remains on the leaves. The numerical response of the predators may be due to the feeding on a combination of other sources available, such as pollen from other plants nearby that could arrive at almond leaves, tetranychid mites (*B. rubrioculus*), or nymphs and larvae of other small arthropods in the trees (e.g., Coccidae), although no direct observation was made. The phytoseiid population in the current study decreased greatly at the beginning of July in most years, when heat is increasing, and relative humidity is low in the Guadalquivir river valley. This behavior has also been observed in coastal citrus orchards in Spain (Ferragut et al. 1987, 1988); however, contrary to what is observed on citrus, in almonds the population increase in autumn is not detectable because almond leaves start to fall in October.

*Typhlodromus* (*A.*) *athenas* is the other phytoseiid species found in the survey, although its densities were low and it was not found in all years. It is reported in various crops and areas of Spain, but always in low density and no special relationship is known with any arthropod (Ferragut et al. 2010). It has been recorded in other countries in the Mediterranean basin on different plants: olive, citrus, cypress, date palm, rosaceous and stone fruit trees, grapes, and others (Papadoulis et al. 2008, cited by Ben Chaaban et al. 2018; Sahraoui et al. 2012). Its ability to control a date mite has been studied in Tunisia (Ben Chaaban et al. 2018).

The other group of natural enemies that could prey on spider mites in the orchard includes insects. The most abundant taxon during the surveys was Chrysopidae, with a constant record of its presence throughout the 4 years of study, but mainly at the egg stage. Chrysopid larvae were rarely observed on leaves, but although important predators of several pest arthropods, they are not considered especially relevant in the control of spider mites and, in particular, of those producing a heavy web (Hoy 2011; Vacante 2016). Various arthropods – coccids, leafhoppers, and others present in the orchard during these 4 years, although always at low densities (Gonzalez-Zamora et al. 2021) – could account for its constant presence. The other two groups of mite predators, although always found in low numbers, were the thrips *S. longicornis* and the coccinellid *Stethorus* sp. *Scolothrips longicornis* has been regularly reported in various crops (e.g., citrus, sweet pepper, nectarines, strawberry) in Spain, feeding on tetranychids that produce a dense web (mainly *T. urticae*) (Lacasa 1993; Lacasa et al. 1993; Sanchez et al. 1995; García-Marí and González-Zamora 1999; Abad et al. 2009), although its efficacy in regulating pest populations was not emphasized. However, some studies (especially under laboratory conditions) have highlighted its good performance, compared positively with *Phytoseiulus persimilis* Athias-Henriot, a specific predator of *T. urticae* (Gerlach and Sengonca 1985) or in different environments and prey (Heidarian et al. 2012; Pakyari and McNeill 2020). In this way, *S. longicornis* has also attracted interest as a promising predator of *S. smirnovi* on almonds in Iran, showing high densities and a quick response to the mite pest densities (Saeidi and Nemati 2020).

*Stethorus* sp. is the other group of potentially relevant predators of tetranychids (Chazeau 1985; Hagen et al. 1999; Biddinger et al. 2009; Hoy 2011) observed on almond leaves in this study. As for *S. longicornis*, its presence was rather scarce and only clearly detected in 2020. *Stethorus punctillum* (Weise) is the species commonly cited in crops in Spain as a spider mite predator (García-Marí and González-Zamora 1999; Alvis et al. 2002; Abad et al. 2009; Martín Gil et al. 2014, 2015).

Mite pest control in the orchard has been based on the use of miticides, although in some years (2017 and 2019) the Tetranychidae population was rather low, but in another year (2018) it was very high, and the application of a miticide drastically reduced its population. On the contrary, in 2020 only one miticide was sprayed in the orchard (see Appendix 1) and the spider mite population reached a significant value in mid-September, with clear damage on leaves (González-Zamora et al. 2021). It was precisely in this year that more *S. longicornis* and *Stethorus* sp. individuals were detected on the leaves. It is generally agreed that these two types of spider mite predators need a certain prey population density in the crop to settle and control the pest population, which can make them appear when damage is done to leaves (García-Marí 2012).

IPM is generally implemented in California almond orchards, following the indications of the California University IPM Guidelines (Strand 2002; Haviland et al. 2022), but although this type of guideline is available in Spain (and in Andalucia; Martín Gil et al. 2015; Durán et al. 2017a), and various natural enemies are identified, they were not generally followed in the almond orchard of this study, where a variety of pesticides and fungicides was used according to the criteria of the advisory technicians. A rational use of pesticides, the use of the ones most compatible with natural enemies, together with the recognition of natural enemies and their role in regulating the pest population, is desirable to improve the biological control of tetranychids in almond and, ultimately, to achieve sustainable agriculture. This study provides new information on the arthropod fauna on almond crops in Spain, being the first long-term study of acarifauna in Spanish almonds.

## Appendix 1

Treatments against pest and diseases in the almond orchard throughout the 4 years of study

**Table:**

Date		Product used	Against
2017	31/01	Copper oxichloride 52%	Diseases
		Paraffinic oil 83%	Eggs and immatures of arthropods
	16–17/02	Thiophanate-methy 70%	Diseases
	1–2/03	Boscalid 26.7% + Pyraclostrobin 6.7%	Diseases
	18–21/03	Boscalid 26.7% + Pyraclostrobin 6.7%	Diseases
	4–6/04	Metconazol 9% Mancozeb 75%	Diseases
		Deltamethrin 2.5%	Aphids
	26/04	Azoxystrobin 25%	Diseases
	16–17/05	Fluopyram 20% + Tebuconazole 20%	Diseases
		Tau-fluvalinate 24%	Aphids, leafhoppers
	9–11/06	Copper oxichloride 52%	Diseases
		Tau-fluvalinate 24% Hexythiazox 10% Abamectin 1.8%	Two-spotted spider mite
	19/08	Thiram 50%	Diseases
	9/09	Imidacloprid 20% Dimethoate 40%	*Monosteira*, *Capnodis* (beetle)
2018	7/03	Fenbuconazole 2.5%	Diseases
	20/03	Tebuconazole 50% + Trifloxystrobin 25%	Diseases
	5/04	Fluxapyroxad 7.5% + Pyraclostrobin 15%	Diseases
		Deltamethrin 2.5%	Aphids
	12/05	Fluopyram 20% + Tebuconazole 20%	Diseases
		Imidacloprid 20%	Aphids, leafhoppers, *Monosteira*, *Capnodis* (beetle)
	9/07	Deltamethrin 2.5%	Lepidoptera, leafhoppers
	21/07	Imidacloprid 20%	Aphids, leafhoppers, *Monosteira*, *Capnodis* (beetle)
	4/09	Thiacloprid 48%	Lepidoptera
		Deltamethrin 2.5%	Lepidoptera, leafhoppers
		Fenpyroximate 5.12%	Two-spotted spider mite
		Mancozeb 75%	Diseases
	5/10	Acetamiprid 20%	Leafhoppers
2019	14/02	Thiophanate-methyl 70% Copper oxichloride 52%	Diseases
	15–17/03	Boscalid 26.7% + Pyraclostrobin 6.7%	Diseases
	04/04	Mancozeb 75% Trifloxystrobin 50%	Diseases
		Deltamethrin 2.5%	Aphids
	12–13/04	Folpet 40% Thiophanate-methyl 70%	Diseases
	27/04	Boscalid 26.7% + Pyraclostrobin 6.7%)	Diseases
	20–21/06	Copper oxichloride 52%	Diseases
		Tau-fluvalinate 24%	Two-spotted spider mite, *Monosteira*, leafhoppers
2020	3–5/03	Metconazole 9% Boscalid 26.7% + Pyraclostrobin 6.7%	Diseases
	17–18/03	Tebuconazole 25% Trifloxystrobin 50%	Diseases
		Deltamethrin 2.5% Acetamiprid 20%	Aphids
	7/04	Thiophanate-methyl 70%	Diseases
	22/04	Difenoconazole 4% + Isopyrazam 10%	Diseases
	10/05	Difenoconazole 25% Azoxystrobin 20% + Cyproconazole 8% Copper oxichloride 52% Mancozeb 75%	Diseases
	20/05	Dodine 40% Mancozeb 75%	Diseases
		Deltamethrin 2.5%	Aphids, leafhoppers
	1/06	Captan 47.5%	Diseases
		Deltamethrin 2.5%	Leafhoppers
	17/06	Mancozeb 75%	Diseases
		Tau-fluvalinate 24% Fenpyroximate 5.12%	Mites, leafhoppers, lepidoptera
	26/08	Copper oxichloride 52%	Diseases
		Deltamethrin 2.5% Acetamiprid 20%	Leafhoppers, lepidoptera

## References

[CR1] Abad R, Pina T, Dembilio Ó (2009). Survey of natural enemies of spider mites (Acari: Tetranychidae) in citrus orchards in eastern Spain. Exp Appl Acarol.

[CR2] Alvis L, Raimundo A, Villalba M, García-Marí F (2002). Identificación y abundancia de coleópteros coccinélidos en cultivos de cítricos valencianos. Bol San Veg Plagas.

[CR3] Barrientos AJ (2004). Curso práctico de entomología.

[CR4] Ben Chaaban S, Chermiti B, Kreiter S (2018) Biology and life-table of *Typhlodromus* (*Anthoseius*) *athenas* (Acari: Phytoseiidae) fed with the Old World Date Mite, *Oligonychus afrasiaticus* (Acari: Tetranychidae). Acarologia 58:52–61. doi: 10.24349/acarologia/20184229

[CR5] Biddinger DJ, Weber DC, Hull LA (2009). Coccinellidae as predators of mites: Stethorini in biological control. Biol Control.

[CR6] Chazeau J (1985) Predaceous insects. In: Spider Mites - Their Biology, Natural Enemies and Control, Volume 1B (ed. by W Helle & MW Sabelis), pp. 211–246. Elsevier, Amsterdam, The Netherlands

[CR7] Chinery M (1997) Guía de campo de los insectos de España y de Europa, 5a ed. Omega, Barcelona

[CR8] Durán JM, Cabello Yuste J, Fernández Gonzalez MI et al (2017a) Plagas y enfermedades del almendro. Secretaria General Técnica. Servicio de Publicaciones y Divulgación., Sevilla (España)

[CR9] Durán JM, Paéz JI, Sánchez AM, Vega JM (2017). Problemática fitosanitaria en las nuevas plantaciones de almendro en la provincia de Sevilla. Phytoma España.

[CR10] EEA (2017) Climate change, impacts and vulnerability in Europe 2016 — European Environment Agency. In: EEA Rep. No 1/2017. https://www.eea.europa.eu/publications/climate-change-impacts-and-vulnerability-2016. Accessed 5 Oct 2021

[CR11] Ferragut F, Costa Comelles J, García-Marí F (1988). Dinámica poblacional del fitoseido *Euseius stipulatus* (Athias-Henriot) y su presa *Panonychus citri* (McGregor) (Acari: Phytoseiidae, Tetranychidae), en los cítricos españoles. Bol Sanid Veg Plagas.

[CR12] Ferragut F, Escudero A (1997). Taxonomía y distribución de los ácaros depredadores del género *Euseius* Wainstein 1962, en España (Acari: Phytoseiidae). Bol Sanid Veg Plagas.

[CR13] Ferragut F, García-Marí F, Costa-Comelles J, Laborda R (1987). Influence of food and temperature on development and oviposition of *Euseius stipulatus* and *Typhlodromus phialatus* (Acari: Phytoseiidae). Exp Appl Acarol.

[CR14] Ferragut F, Pérez-Moreno I, Iraola V, Escudero A (2010). Ácaros depredadores en las plantas cultivadas-Familia Phytoseiidae.

[CR15] Ferragut F, Santonja MC (1989). Taxonomia y distribucion de los acaros del genero *Tetranychus* Dufour 1832 (Acari: Tetranychidae), en Espanña. Bol Sanid Veg Plagas.

[CR16] García-Marí F (2012). Plagas de los cítricos-Gestión integrada en paises de clima mediterráneo.

[CR17] García-Marí F, González-Zamora JE (1999). Biological control of *Tetranychus urticae* (Acari: Tetranychidae) with naturally occurring predators in strawberry plantings in Valencia, Spain. Exp Appl Acarol.

[CR18] García-Tejero IF, Lipan L, Gutiérrez-Gordillo S (2020). Deficit Irrigation and Its Implications for HydroSOStainable Almond Production. Agronomy.

[CR19] Gerlach S, Sengonca Ç (1985). Comparative studies on the effectiveness of the predatory mite, *Phytoseiulus persimilis* Athias-Henriot, and the predatory thrips, *Scolothrips longicornis* Priesner. J Plant Dis Prot.

[CR20] González-Zamora JE, Ruiz-Aranda C, Rebollo-Valera M (2021). Deficit Water Irrigation in an Almond Orchard Can Reduce Pest Damage. Agronomy.

[CR21] Grafton-Cardwell EE, Bentley W, Bianchi M (2020). Surveys of 12 California crops for phytoseiid predatory mites show changes compared to earlier studies. Calif Agric.

[CR22] Gutiérrez-Gordillo S, Durán-Zuazo VH, García-Tejero IF (2019). Response of three almond cultivars subjected to different irrigation regimes in Guadalquivir river basin. Agric Water Manag.

[CR23] Hagen KS, Mills NJ, Gordh G, McMurtry JA(1999) Terrestrial arthropod predators of insect and mite pests. Handbook of Biological Control – Principles and Applications of Biological Control (ed. by TS Bellows & TW Fisher), pp. 383–504. Academic Press, San Diego, CA, USA

[CR24] Haviland DR, Rijal JP, Rill SM (2021). Management of Navel Orangeworm (Lepidoptera: Pyralidae) Using Four Commercial Mating Disruption Systems in California Almonds. J Econ Entomol.

[CR25] Haviland DR, Rill SM, Gordon CA (2021). Field Biology of *Scolothrips sexmaculatus* (Thysanoptera: Thripidae) as a Predator of *Tetranychus pacificus* (Acari: Tetranychidae) in California Almonds. J Econ Entomol.

[CR26] Haviland DR, Rill SM, Gordon CA (2021). Evaluation of Sticky Traps for Monitoring *Scolothrips sexmaculatus* (Thysanoptera: Thripidae) and *Stethorus punctum* (Coleoptera: Coccinellidae) as Predators of Spider Mites in California Almonds. J Econ Entomol.

[CR27] Haviland DR, Rill SM, Gordon CA (2021). Treatment Thresholds for *Tetranychus pacificus* (Acari: Tetranychidae) in California Almonds Based on Monitoring for the Natural Enemy *Scolothrips sexmaculatus* (Thysanoptera: Thripidae). J Econ Entomol.

[CR28] Haviland DR, Symmes E, Adaskaveg J et al(2022) UC IPM Pest Management Guidelines: Almond. https://www2.ipm.ucanr.edu/agriculture/almond/. Accessed 10 Jan 2022

[CR29] Heidarian M, Fathipour Y, Kamali K (2012). Functional response, switching, and prey-stage preference of *Scolothrips longicornis* (Thysanoptera: Thripidae) on *Schizotetranychus smirnovi* (Acari: Tetranychidae). J Asia Pac Entomol.

[CR30] Hoy MA (2011). Agricultural Acarology: Introduction to integrated mite management.

[CR31] Hoy MA, Roush R, Smith K, Barclay L (1979). Spider mites and predators in San Joaquin Valley almond orchards. Calif Agric.

[CR32] Jeppson LR, Keifer HH, Baker EL (1975). Mites injurious to economic plants.

[CR33] Junta de Andalucia (2016) Caracterización del sector de la almendra en Andalucía. https://www.juntadeandalucia.es/export/drupaljda/estudios_informes/16/12/Caracterización del sector de la almendra_0.pdf. Accessed 21 Oct 2021

[CR34] Lacasa A(1993) Importancia de los trips (Insecta: Thysanoptera) en la Agricultura española. In: Bhatti B (ed) Advances in Thysanopterology, Journal of Pure and Applied Zoology. pp 267–285

[CR35] Lacasa A, Martínez C, Torres J(1993) *Frankliniella occidentalis* (Perg.) en los cultivos de nectarina de Murcia. Evolución de las poblaciones y comportamiento de variedades. Boletín Sanid Veg Plagas 19:335–344

[CR36] MAPA (2015) Superficie y producción de almendro 2014. https://www.mapa.gob.es/estadistica/pags/anuario/2015/TABLASPDF/CAPITULO13/pdfc13_10.1.2.pdf. Accessed 21 Oct 2021

[CR37] MAPA (2020) Avance de superficie y producción de almendro 2020. https://www.mapa.gob.es/es/estadistica/temas/estadisticas-agrarias/agricultura/superficies-producciones-anuales-cultivos/. Accessed 21 Oct 2021

[CR38] MAPA (2019) Serie histórica almendro 2014–2018. https://www.mapa.gob.es/estadistica/pags/anuario/2019/CAPITULOSPDF/CAPITULO07/pdfc07_10.1.1.pdf. Accessed 21 Oct 2021

[CR39] Martín-Palomo MJ, Corell M, Girón I (2019). Limitations of using trunk diameter fluctuations for deficit irrigation scheduling in almond orchards. Agric Water Manag.

[CR40] Martín Gil A, Arrivas Carrasco G, Barrios Sanroma G (2015). Guía de gestión integrada de plagas: Almendro. Secretaria General Técnica.

[CR41] Martín Gil A, Lozano Tomás CM, Batllori Obiol L (2014). Guía de gestión integrada de plagas: Frutales de pepita. Secretaria General Técnica.

[CR42] McMurtry JA, Croft BA (1997). Life-styles if phytoseiid mites and their roles in biological control. Annu Rev Entomol.

[CR43] McMurtry JA, Moraes GJ, Sourassou NF (2013). Revision of the lifestyles of phytoseiid mites (Acari: Phytoseiidae) and implications for biological control strategies. Syst Appl Acarol.

[CR44] Mound LA, Morison GD, Pitkin BR, Palmer JM (1976). Thysanoptera.

[CR45] Ollero-Lara A, Agustí-Brisach C, Lovera M (2019). Field susceptibility of almond cultivars to the four most common aerial fungal diseases in southern Spain. Crop Prot.

[CR46] Ollero-Lara A, Lovera Manzanares M, Roca Castillo LF (2016). Susceptibilidad varietal del almendro a la mancha ocre en Andalucía. Vida Rural.

[CR47] Pakyari H, McNeill MR (2020). Effects of photoperiod on development and demographic parameters of the predatory thrips *Scolothrips longicornis* fed on *Tetranychus urticae*. Bull Entomol Res.

[CR48] Papadoulis G, Th, Emmanouel NG, Kapaxidi EV (2008). Phytoseiidae of Greece and Cyprus.

[CR49] Rice RE, Jones RA(1978) Mites in almonds and stone fruits.Calif Agric20–21

[CR50] Saeidi Z, Nemati A (2020). Almond spider mite, *Schizotetranychus smirnovi* (Acari: Tetranychidae): population parameters in laboratory and field conditions. Persian J Acarol.

[CR51] Sahraoui H, Lebdi Grissa K, Kreiter S (2012). Phytoseiid mites (Acari: Mesostigmata) of Tunisian citrus orchards: Catalogue, biogeography and key for identification. Acarologia.

[CR52] Sánchez-Ramos I, Pascual S, Fernández CE (2015). Effect of temperature on the survival and development of the immature stages of *Monosteira unicostata* (Hemiptera: Tingidae). Eur J Entomol.

[CR53] Sanchez JA, Contreras J, Lacasa A, Llorca M (1995). Datos preliminares sobre la utilización de *Orius laevigatus* (Fieber) en el control de *Frankliniella occidentalis* (Pergande) en pimiento en invernadero. Phytoma España.

[CR54] Shorey HH, Gerber RG (1996). Use of Puffers for Disruption of Sex Pheromone Communication Among Navel Orangeworm Moths (Lepidoptera: Pyralidae) in Almonds, Pistachios, and Walnuts. Environ Entomol.

[CR55] Strand L(2002) Integrated pest management for almonds, 2nd editio. University of California, statewide integrated pest management project. Division of Agriculture and Natural Resources, Oakland, CA

[CR56] Tollerup K, Higbee B (2020). Evaluation of a ‘Preventative’ Strategy to Manage Spider Mites on Almond. Insects.

[CR57] Torguet Pomar L, Batlle Caravaca I, Alegre S, Miarnau i Prim X (2016). Nuevas plagas y enfermedades emergentes, una amenaza para el cultivo del almendro en España. Rev Frutic.

[CR58] Vacante V(2016) The handbook of mites of economic plants-Identification, bio-ecology and control, First ed. CAB International, Wallinford (Oxfordshire)

[CR59] Welter SC, Barnes MM, Ting IP, Hayashi JT (1984). Impact of Various Levels of Late-Season Spider Mite (Acari: Tetranychidae) Feeding Damage on Almond Growth and Yield. Environ Entomol.

[CR60] Wilson H, Burks CS, Reger JE, Wenger JA (2020). Biology and Management of Navel Orangeworm (Lepidoptera: Pyralidae) in California. J Integr Pest Manag.

[CR61] Wilson LT, Hoy MA, Zalom FG, Smilanick JM (1984). Sampling mites in almonds: I. Within-tree distribution and cumping pattern of mites with comments on predatory-prey interactions. Hilgardia.

